# The role of WTAP in regulating macrophage-mediated osteoimmune responses and tissue regeneration in periodontitis

**DOI:** 10.3389/fimmu.2024.1423378

**Published:** 2024-07-16

**Authors:** Yuman Li, Yue Yang, Yuting Niu, Yao Li, Zhewen Hu, Shiyu Sun, Yiming Chen, Bo Hu, Ying Huang, Xuliang Deng

**Affiliations:** ^1^ Department of Geriatric Dentistry, Peking University School and Hospital of Stomatology & National Center for Stomatology & National Clinical Research Center for Oral Diseases & National Engineering Research Center of Oral Biomaterials and Digital Medical Devices, Beijing, China; ^2^ Department of Prosthodontics, The First Clinical Division, Peking University School and Hospital of Stomatology & National Center for Stomatology & National Clinical Research Center for Oral Diseases & National Engineering Research Center of Oral Biomaterials and Digital Medical Devices, Beijing, China

**Keywords:** periodontitis, macrophage, WTAP, osteoimmune, tissue regeneration

## Abstract

Periodontitis, delineated by the destruction of structures that support teeth, is predominantly propelled by intricate immune responses. Immunomodulatory treatments offer considerable promise for the management of this ailment; however, the modulation of the periodontal immune microenvironment to facilitate tissue regeneration presents a substantial biomedical challenge. Herein, our study investigates the role of Wilms’ tumor 1-associating protein (WTAP), a critical m^6^A methyltransferase, in the immunomodulation of periodontitis and assesses its viability as a therapeutic target. We observed heightened expression of WTAP in macrophages extracted from gingival tissues impacted by periodontitis, with a strong association with M1 polarization. Via loss-of-function experiments, we demonstrated that diminishing WTAP expression precipitates a transition from M1 to M2 macrophage phenotypes amidst inflammatory conditions, thus improving the periodontal immune landscape. Further, RNA sequencing and indirect co-culture assays indicated that suppressing of WTAP expression modulates osteoimmune responses and enhances the osteogenic differentiation of bone marrow stromal cells. The local deployment of adeno-associated virus-shWTAP in murine models of periodontitis robustly validated the therapeutic promise of targeting WTAP in this disease. Collectively, our findings highlight the crucial role of WTAP in orchestrating macrophage-mediated osteoimmune responses and tissue regeneration in periodontitis, proposing novel avenues for immunotherapeutic interventions in its treatment.

## 1 Introduction

Periodontitis, a pervasive global health concern, represents a chronic inflammatory disease that detrimentally affects the supportive structures surrounding teeth and poses significant health and economic burdens (1–3). Although traditional periodontal therapies, such as local mechanical debridement and antibiotic treatment, are effective in managing localized infections, they frequently fail to regenerate periodontal tissues (4). Research suggests that while dental plaque biofilms initiate periodontitis, the progression and destruction of the disease are chiefly propelled by the host’s immune responses (5, 6). Hence, effectively modulating these immune responses may be key to improving periodontal tissue regeneration.

Macrophages, central to coordinating immune responses within periodontal regions, exhibit notable plasticity (7–9). They play critical roles in both the onset and resolution of periodontitis by adopting pro-inflammatory (M1) and anti-inflammatory (M2) phenotypes in reaction to environmental cues (7–9). These phenotypes exert divergent effects on inflammation and tissue repair processes. The equilibrium between M1 and M2 macrophages is vital for maintaining periodontal health; however, periodontitis is characterized by a marked shift towards M1 polarization. This alteration results in an elevated M1/M2 ratio and the secretion of pro-inflammatory cytokines, contributing to exacerbated inflammation and alveolar bone loss (10, 11). Addressing this imbalance by promoting a shift in macrophage polarization from M1 to M2 under inflammatory conditions is viewed as a potential strategy for tissue regeneration, although this transition presents substantial challenges.

Recent studies have highlighted the significance of N6-methyladenosine (m^6^A), the most ubiquitous epigenetic modification found in eukaryotic messenger RNA (mRNA), as an instrumental factor in macrophage polarization (12–14). Wilms’ tumor 1-associating protein (WTAP), an essential regulatory subunit of the m^6^A methyltransferase complex, which includes METTL3 and METTL14, is integral to RNA modification and the regulation of gene expression (15). While WTAP’s impact has been thoroughly investigated across various physiological and pathological contexts (16–19), its role in facilitating periodontal tissue regeneration by modulating macrophage polarization is still not well understood.

In this study, we consistently observed elevated WTAP expression in macrophages derived from gingival tissues of both humans and mice afflicted with periodontitis, with a strong correlation to M1 polarization. Inhibition of WTAP promoted a shift toward the M2 phenotype in macrophages within inflammatory environments, thereby cultivating an immune milieu favorable to tissue regeneration. RNA sequencing revealed that reducing WTAP expression modulates the macrophage-mediated osteoimmune responses and augments osteogenesis. Additionally, indirect co-culture experiments with bone marrow stromal cells (BMSCs) and macrophages demonstrated that diminishing WTAP expression enhances osteogenic differentiation in BMSCs. Furthermore, *in vivo* experiments using adeno-associated virus (AAV)-shWTAP to silence WTAP in macrophages validated its potential to promote tissue regeneration in periodontitis. Collectively, our findings underscore the critical role of WTAP in orchestrating macrophage-mediated osteoimmune responses and tissue regeneration in periodontitis, presenting a promising target for therapeutic intervention.

## 2 Materials and methods

### 2.1 Mice

C57BL/6 wild type mice were acquired from Beijing Vital River Laboratory Animal Technology Co., Ltd. (Beijing, China). All animals were housed under specific pathogen-free conditions, maintained on a 12:12-hour light/dark cycle, at a constant temperature of 24 ± 0.5°C and 40–70% relative humidity. The animal study protocols were approved by the Ethics Committee of Peking University Health Science Center (Approved number: PUIRB-LA2023433).

### 2.2 Ligature-induced periodontitis model

Experimental periodontitis was induced in mice as previously described (20). Briefly, 5–0 silk ligatures were placed around the bilateral maxillary second molars of the mice for 14 days to induce periodontitis. Mice in the control group did not receive ligature placement. At the indicated endpoints, mice were euthanized, and bilateral maxillary tissues were collected for subsequent analyses.

### 2.3 Quantitative real-time polymerase chain reaction

Total RNA was extracted from tissues and cells using Trizol reagent (Invitrogen) and then reverse-transcribed into cDNA using Evo M-MLV RT Premix (AG11706, Accurate Biotechnology), according to the manufacturer’s instructions. RT-PCR was conducted using the Hieff UNICON^®^ Universal Blue qPCR SYBR Green Master Mix (11184ES08, Yeasen) with specific primers. Gene expression levels were normalized to *gapdh* mRNA levels. Primer sequences were listed in **Supplementary Table 1**.

### 2.4 Histological analysis

Mouse maxillae were dissected, fixed with 4% paraformaldehyde, decalcified in EDTA solution, dehydrated in a graded series of alcohol, paraffin embedded and sectioned into 5 µm slices for histological analysis. Slices were stained with Hematoxylin and Eosin (H&E) to assess general tissue morphology. For tissue immunofluorescence staining, slices were deparaffinized, underwent antigen retrieval, and were permeabilized and blocked. Subsequently, slices were incubated with primary antibodies overnight at 4°C, followed by secondary antibodies for 1 hour in darkness at room temperature, and counterstained with 4′,6-diamidino-2-phenylindole (DAPI). Primary antibodies included anti-WTAP (60188–1-lg, Proteintech), anti-CD68 (ab125212, Abcam), anti-CD206 (ab64693, Abcam), and anti-CD86 (13395–1-AP, Proteintech). Secondary antibodies included donkey anti-mouse IgG H&L Alexa Fluor 647 (A-31571, Invitrogen), and donkey anti-rabbit IgG H&L Alexa Fluor 594 (A-21207, Invitrogen). Images were captured using a 3DHISTECH digital slice scanner (3DHISTECH, Hungary).

### 2.5 Isolation, induction and immortalization of bone marrow-derived macrophages

Bone marrow cells were harvested from the femurs and tibiae of 8-week-old C57BL/6 mice and cultured in Dulbecco’s Modified Eagle Medium (DMEM, Procell, China) supplemented with 20 ng/ml granulocyte-macrophage colony-stimulating factor (GM-CSF, Peprotech, USA). This culture was maintained for 7 days to induce differentiation into bone marrow-derived macrophages (BMDMs). The BMDMs were incubated at 37°C in a humidified atmosphere containing 5% CO2. For some experiments, BMDMs were exposed to an inflammatory environment simulated by the addition of lipopolysaccharide (LPS).

To immortalize the BMDMs, an SV40-overexpressing lentivirus was created using the EF1α-SV40-IRES-puromycin vector. BMDMs were seeded in a 6-well plate and allowed to adhere overnight. The following day, the medium containing the SV40-overexpressing lentivirus was added. After 12 hours, the culture was inspected, and the medium was replaced with fresh complete culture medium. Upon reaching approximately 90% confluence, the BMDMs underwent successive passaging and continued cultivation.

### 2.6 WTAP gene knockdown

To knock down the expression of WTAP in BMDMs, we used specific shRNA sequences targeting the gene. The shRNA sequence used for WTAP knockdown was as follows: 5’-CGAAGAACCTCTTCCTAAAA-3’. A non-targeting shRNA sequence was used as a control. BMDMs were transfected with WTAP-targeting shRNA lentiviral particles (shWTAP) or control shRNA lentiviral particles (shNC) using Lipofectamine 3000 (Invitrogen) according to the manufacturer’s instructions. After 48 hours of transfection, the cells were cultured in complete medium supplemented with 2 µg/mL puromycin for 72 hours to select successfully transfected cells. The efficiency of gene knockdown was confirmed by RT-PCR. These selected cells were then used for downstream experimental analyses to ensure the purity and reliability of the results.

### 2.7 Macrophage polarization under inflammatory conditions

To verify the effects of WTAP on macrophage polarization within an inflammatory setting, porphyromonas gingivalis-lipopolysaccharide (LPS) was selected as the stimulus to mimic periodontitis conditions. BMDMs treated with shNC and shWTAP were cultured in 6-well plates until they reached approximately 80% confluence. The cells were then stimulated with 1 µg/mL porphyromonas gingivalis-LPS for 24 hours. Subsequently, the expression of M1 and M2 phenotype markers in BMDMs was analyzed.

### 2.8 Quantification of the m^6^A modification

Total RNA was isolated using Trizol reagent (Invitrogen) in accordance with the manufacturer’s instructions. The relative m^6^A content in total RNA was measured using the EpiQuik m^6^A RNA Methylation Quantification Kit (Colorimetric) (P- 9005–48, Epigentek), following the manufacturer’s guidelines. For the assay, 200 ng of RNA from each sample was dispensed into the designated wells of a 96-well plate, with all assays conducted in triplicate. The procedure included both negative and positive controls, along with a standard curve ranging from 0.02 ng to 1 ng of m^6^A, as recommended by the kit instructions. Subsequently, the capture antibody solution and the detection antibody solution were added sequentially to the assay wells in specified dilutions. The m^6^A levels were quantified colorimetrically by measuring the absorbance at 450 nm. Data analysis was then performed based on the established standard curve, ensuring accuracy in the quantification of m^6^A levels within the samples.

### 2.9 Immunofluorescence cell staining

Cells were gently rinsed with phosphate-buffered saline (PBS) and fixed in 4% paraformaldehyde for 15 minutes. Permeabilization was achieved using 0.5% Triton X-100 (Sigma) for 5 minutes at room temperature. Cells were then blocked with 3% bovine serum albumin (BSA, Solarbio) for 30 minutes before overnight incubation at 4°C with primary antibodies, including anti-CD206 (ab64693, Abcam) and anti-CD86 (13395–1-AP, Proteintech). Following primary antibody incubation, cells were treated with goat anti-rabbit IgG H&L Alexa Fluor 488 (A-11008, Invitrogen) for 1 hour in the dark at room temperature. F-actin and nuclei were stained with TRITC phalloidin (CA1610, Solarbio) and DAPI (Sigma), respectively. Images were acquired using a confocal laser scanning microscope (Leica, Germany).

### 2.10 Flow cytometry

Cultured cells were labeled with FITC-conjugated anti-mouse CD206 antibody (141704, BioLegend) and APC-conjugated anti-mouse CD86 antibody (105012, BioLegend) and incubated for 30 minutes away from light. Cells were then centrifuged, washed with PBS, and the pellet was resuspended for analysis. Flow cytometric data were acquired using a Calibur2 flow cytometer (BD, USA) and analyzed with FlowJo software.

### 2.11 Bulk RNA-seq

Total RNA was extracted from BMDMs treated with shNC and shWTAP using Trizol reagent (Invitrogen) according to the manufacturer’s instructions. The RNA samples were subsequently submitted to BGI Genomics Co., Ltd (Shenzhen, China) for sequencing analysis. Libraries were prepared and sequenced on the BGISEQ platform utilizing a SE50 strategy. Sequencing data were processed with SOAPnuke to obtain clean reads, which were stored in FASTQ format. Data analysis and mining were conducted using the Dr. Tom Multi-omics Data mining system (https://biosys.bgi.com) and OmicStudio tools (https://www.omicstudio.cn/tool). Differentially expressed genes (DEGs) were identified with thresholds of |log2FC| ≥ 1 and *P*-value < 0.05.

### 2.12 Osteogenesis evaluation

Macrophage-conditioned medium was prepared using the supernatants from shNC- and shWTAP-treated BMDMs, and mixed with osteogenic induction medium for mouse BMSCs (MUXMX-90021, Cyagen) at a ratio of 1:2. The expression of osteogenic genes, including *runt-related transcription factor 2 (runx2), bone morphogenetic protein 2 (bmp2), osteocalcin (ocn), osteopontin (opn), and osteoprotegerin (opg)*, in BMSCs cultured in this conditioned medium over periods of 4, 7, and 14 days was quantified via RT-PCR. Alkaline phosphatase (ALP) staining and Alizarin Red-S (ARS) staining were performed on days 14 and 21 of culture, respectively. For ALP staining, cells were fixed in 4% paraformaldehyde for 15 minutes and stained using BCIP/NBT alkaline phosphatase color development kits (C3206, Beyotime). ALP activity was quantified using ALP assay kits (P0321S, Beyotime). For ARS staining, cells were fixed and stained with alizarin red (Cyagen) for 15 minutes. After rinsing with deionized water, calcium nodules were observed microscopically. Stained samples were then solubilized in cetylpyridinium chloride (CPC) and the absorbance at 562 nm was measured using a spectrophotometer to quantify mineralization.

### 2.13 Micro-computed tomography analysis

Maxillary samples were fixed in 4% paraformaldehyde overnight and subsequently scanned with Inveon microtomography system (Siemens, Munich, Germany). The specific scanning parameters were set as follows: voltage of 60 kVp, current of 220 μA, exposure time of 1500 ms per frame, voxel size of 10 µm, and 360 projections with 1-degree step rotation.

After scanning, 3D reconstruction and image analysis were conducted using Siemens Inveon Acquisition Workplace (IAW) software. The region of interest (ROI) included the alveolar bone surrounding the second molar to assess bone mineral density (BMD). Bone loss was quantified by measuring the distance from the cementoenamel junction (CEJ) to the alveolar bone crest (ABC) in the reconstructed 3D images.

### 2.14 Production of AAV for silence of WTAP in mouse macrophages

To construct AAV vectors for the specific silence of WTAP in mouse macrophages, we first selected target sequences for mouse WTAP mRNA using the Dharmacon siDESIGN center (http://www.dharmacon.com). A non-specific control shRNA sequence (shNC) was also designed to serve as a negative control, ensuring no significant sequence similarity to any known mouse genes. These sequences were cloned under the control of the CD68 promoter, which is highly active in macrophages, and a U6 promoter into an AAV vector backbone to drive shRNA expression. For virus production, the recombinant plasmids were co-transfected with helper plasmids into HEK293 cells using Lipofectamine 3000 transfection reagent (L3000150, Invitrogen). The helper plasmids provided necessary AAV rep and cap genes, as well as adenoviral helper functions. The virus-containing medium was harvested 72 hours post-transfection, and the virus particles were purified through gradient ultracentrifugation. The titer of the AAV particles was determined by AAV Quantitation Titer Kit (Cell Biolabs, San Diego, CA, USA).

### 2.15 AAV-shWTAP transduction of periodontitis mice

Following 14 days of silk ligation-induced periodontitis, mice were relieved from silk ligatures and subsequently administered with 3 μL (2x10^9^ packaged viral particles in PBS) of either AAV-shWTAP or AAV-shNC viral vectors. Injections were performed using a 5-μL Hamilton syringe inserted approximately 0.3–0.5 mm below the gingival margin of the maxillary molars on both the right and left palatal sides. Mice in the negative control group (normal) did not undergo silk ligation or receive AAV treatment.

### 2.16 Single cell RNA sequencing analysis

Single-cell RNA sequencing data previously published by Williams et al. (21) were retrieved as raw files from the GEO database. These files were processed and transformed into Seurat-compatible objects for further analysis. All downstream bioinformatic analyses were performed using Omicsmart, a dynamic real-time interactive online platform for data analysis (http://www.omicsmart.com).

### 2.17 Statistical analysis

Statistical analysis was performed using GraphPad Prism 8.0.2 software (GraphPad Software Inc., San Diego, CA, USA). Differences between two groups were evaluated using two-tailed unpaired Student’s t-test, while comparisons among three or more groups were analyzed using one-way ANOVA. Data were presented as mean ± standard deviation (SD), and *P* < 0.05 was considered statistically significant.

### 2.18 Illustration of schematic diagrams

The schematic diagrams were generated utilizing Figdraw (https://www.figdraw.com/).

## 3 Results

### 3.1 Enhanced expression of WTAP in macrophages correlates with a pro-inflammatory phenotype in periodontitis

**Figure 1:**
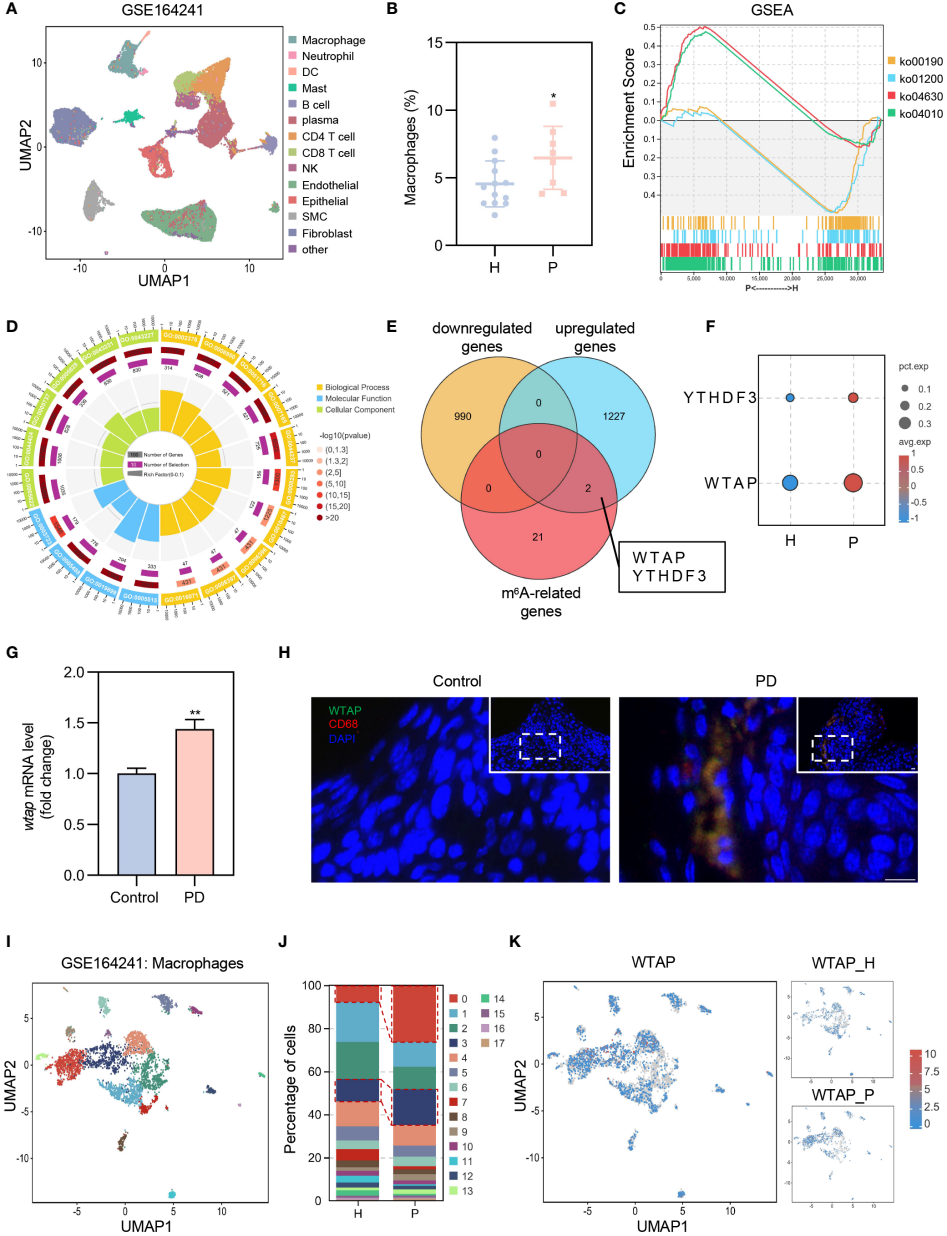
Elevated WTAP expression in macrophages links to a pro-inflammatory phenotype in periodontitis. **(A)** UMAP visualization of 13 cell clusters derived from GEO database (GSE164241), including macrophages, neutrophils, dendritic cells (DCs), mast cells, B cells, plasma cells, CD4^+^ T cell, CD8^+^ T cell, natural killer (NK) cells, endothelial cells, epithelial cells, smooth muscle cells (SMC) and fibroblasts. **(B)** Comparison of the proportion of gingival macrophages between patients with periodontitis (P) and healthy control (H) using scRNA-seq data (H, n = 13; P, n = 8). **(C)** GSEA of genes expressed in macrophages of patients with periodontitis (P) versus healthy subjects (H). ko04010: MAPK signaling pathway; ko04630: JAK-STAT signaling pathway; ko00190: oxidative phosphorylation; ko01200: carbon metabolism. **(D)** GO enrichment analysis of genes that are differentially upregulated in macrophages of patients with periodontitis. **(E)** Three-way Venn diagram displaying the overlap and unique counts of differentially expressed genes in macrophages from GSE164241 dataset and m^6^A-related genes: yellow for genes downregulated in periodontitis, blue for genes upregulated in periodontitis, red for m^6^A-related genes. **(F)** Dot plot illustrating the expression of YTHDF3 and WTAP in macrophages in patients with periodontitis (P) and healthy control (H). Dot color represents average expression level; dot size indicates the percentage of cells expressing the gene. **(G)** RT-PCR analysis comparing *wtap* mRNA level in gingival tissues from control and periodontitis (PD) mice (n = 3). **(H)** Representative immunofluorescent staining of WTAP (green), CD68 (red) and nuclei (DAPI, blue) in gingival tissue sections. Scale bar = 10 μm. **(I)** Subclustering UMAP plot displaying macrophage heterogeneity in patients with periodontitis (P) and healthy control **(H)**, colored by cell type. **(J)** Proportions of the 18 distinct macrophage subclusters in patients with periodontitis (P) and healthy control (H). **(K)** UMAP plot showing WTAP expression across macrophage subclusters in patients with periodontitis (P) and healthy control (H). Data are presented as the mean ± SD from at least three independent experiments. *P* values were calculated using two-tailed Student’s t test (**B, G**); *P <0.05, **P <0.01.

Periodontitis is an inflammatory disease characterized by the dysregulation of the periodontal immune responses. To systematically assess the immune status in periodontitis, we analyzed published single-cell RNA sequencing (scRNA-seq) data from both healthy adults and individuals with periodontitis (21). We performed joint clustering of all cells within the Uniform Manifold Approximation and Projection (UMAP) space and identified 13 major cell types using canonical marker genes (**Figure 1A**; **Supplementary Figure 1**). Notably, the proportion of macrophages was significantly higher in the periodontitis group (**Figure 1B**). Gene Set Enrichment Analysis (GSEA) revealed that genes upregulated in macrophages from periodontitis patients were predominantly associated with the “MAPK signaling pathway (ko04010)” and “JAK-STAT signaling pathway (ko04630)”, while downregulated genes were primarily linked to “oxidative phosphorylation (ko00190)” and “carbon metabolism (ko01200)” (**Figure 1C**). Additionally, genes related to gene ontology (GO) terms “immune system process (GO:0002376)”, “response to stress (GO:0006950)”, “RNA processing (GO:0006396)”, and “mRNA processing (GO:0006397)” were overrepresented in the significantly upregulated genes in macrophages from patients with periodontitis compared to healthy controls (**Figure 1D**). These findings suggest a pro-inflammatory phenotype of gingival macrophages in periodontitis, potentially linked to enhanced RNA processing, wherein m^6^A modifications play a crucial role. To elucidate key m^6^A regulators associated with this pro-inflammatory phenotype, we conducted a correlation analysis between differentially expressed genes in macrophages and m^6^A-related genes. Notably, two m^6^A-related genes, WTAP and YTHDF3, showed higher expression in macrophages from periodontitis patients compared to healthy subjects (**Figure 1E**). Among these, WTAP displayed significantly higher expression levels and pronounced differences, warranting further investigation (**Figure 1F**).

To validate the impact of periodontitis on WTAP expression, we established a ligature-induced periodontitis model over 14 days. RT-PCR confirmed a significant upregulation of *wtap* in gingival tissues of periodontitis mice (**Figure 1G**). Immunofluorescence assays demonstrated a marked increase in WTAP expression within macrophages in these tissues (**Figure 1H**). Further unsupervised clustering analysis of macrophages identified 18 subclusters, with subclusters C0 and C3 showing notable increases in periodontitis patients (**Figures 1I, J**). The upregulated genes in C0 and C3 were associated with KEGG signaling pathways linked to the M1 polarization phenotype of macrophages, indicating that these subclusters consist of pro-inflammatory macrophages (**Supplementary Figure 2**). Moreover, WTAP expression in C0 and C3 was significantly elevated in patients with periodontitis compared to healthy controls (**Figure 1K**; **Supplementary Figure 3**). Collectively, these results underscore that WTAP induction in periodontitis is predominantly observed in macrophages, and its elevated expression may significantly contribute to the macrophage M1 polarization phenotype associated with this disease.

### 3.2 WTAP regulates macrophage polarization under inflammatory conditions

**Figure 2:**
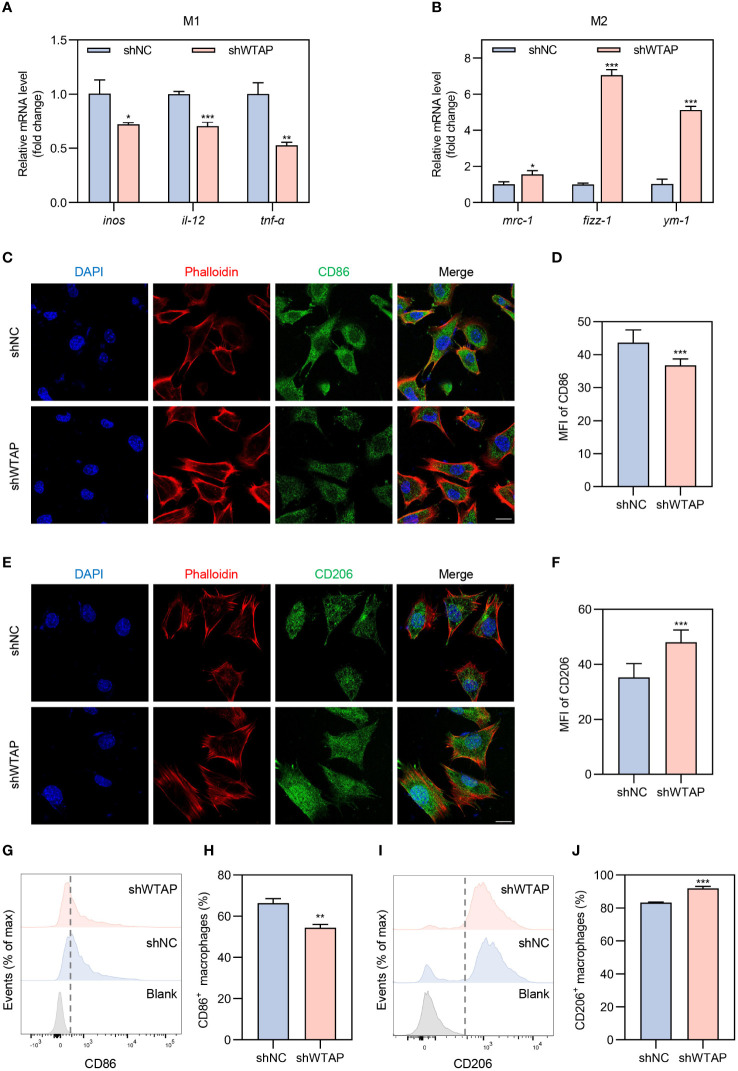
Modulation of macrophage polarization towards the M2 phenotype by WTAP knockdown under inflammatory conditions. **(A)** RT-PCR analysis quantifying the mRNA levels of pro-inflammatory markers *inos*, *il-12* and *tnf-α* in BMDMs from the shNC and shWTAP groups (n = 3). **(B)** RT-PCR analysis quantifying the mRNA levels of anti-inflammatory markers *mrc-1*, *fizz-1* and *ym-1* in BMDMs from the shNC and shWTAP groups (n = 3). **(C)** Representative immunofluorescent staining of BMDMs showing CD86 (green), phalloidin (red) and nuclei (DAPI, blue). Scale bar = 20 μm. **(D)** Quantitative analysis of the mean fluorescence intensity (MFI) of CD86 in BMDMs from the shNC and shWTAP groups. **(E)** Representative immunofluorescent staining of BMDMs showing CD206 (green), phalloidin (red) and nuclei (DAPI, blue). Scale bar = 20 μm. **(F)** Quantitative analysis of the MFI of CD206 in BMDMs from the shNC and shWTAP groups. **(G, H)** Flow cytometry analysis of CD86 expression levels **(G)** and quantitative analysis of the percentage of CD86^+^ macrophages **(H)** in BMDMs from the shNC and shWTAP groups (n = 3). **(I, J)** Flow cytometry analysis of CD206 expression levels **(I)** and quantitative analysis of the percentage of CD206^+^ macrophages **(J)** in BMDMs from the shNC and shWTAP groups (n = 3). Data are presented as the mean ± SD from at least three independent experiments. *P* values were calculated using two-tailed Student’s t test; *P <0.05, **P <0.01, ***P <0.001.

To delve deeper into the role of WTAP in macrophage polarization within an inflammatory setting, we transfected BMDMs with WTAP-specific knockdown lentivirus. Following transfection, these cells were exposed to a LPS-induced inflammatory environment to mimic inflammation-related stress. The efficacy of WTAP knockdown was verified by RT-PCR, which demonstrated a significant reduction in *wtap* expression (**Supplementary Figure 4**). We then evaluated the impact of WTAP knockdown on m^6^A methylation levels in BMDMs under these inflammatory conditions. Our findings indicated a substantial decrease in m^6^A methylation levels post-knockdown (**Supplementary Figure 5**). RT-PCR analysis revealed that, compared to the shNC control group, there was a significant reduction in the expression of classical M1 markers such as *inos*, *il-12*, and *tnf-α* in the shWTAP-treated BMDMs (**Figure 2A**). In contrast, the expression of M2 markers, including *mrc-1*, *fizz-1*, and *ym-1*, was markedly increased (**Figure 2B**). Immunofluorescence staining corroborate these findings, showing a decrease in CD86 expression and an increase in CD206 expression in the shWTAP group relative to controls (**Figures 2C–F**). Flow cytometry confirmed these observations, indicating decreased CD86 and increased CD206 expression in the shWTAP group compared to the shNC group (**Figures 2G–J**). These results collectively suggest that the suppression of WTAP in macrophages induces a shift from M1 to M2 polarization under inflammatory conditions, highlighting WTAP’s significant regulatory role in modulating macrophage functional phenotypes. This shift could be pivotal for therapeutic strategies aimed at modulating immune responses in periodontitis.

### 3.3 Transcriptomic shifts in macrophages induced by WTAP knockdown modulate osteoimmune responses and promote osteogenesis

**Figure 3:**
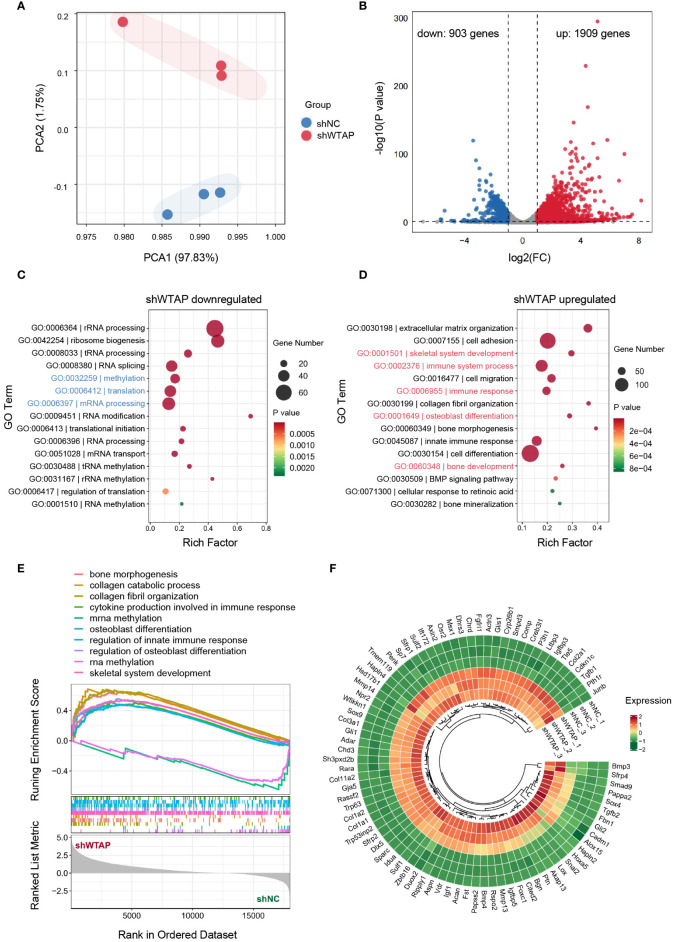
Transcriptomic shifts in macrophages due to WTAP knockdown enhance osteoimmune response and osteogenesis. **(A)** PCA of the normalized RNA-seq abundance data from BMDMs treated with either shNC or shWTAP (n = 3 per group). **(B)** Volcano plots analysis identifying the differentially expressed genes (DEGs) in BMDMs between the shWTAP and shNC groups. Genes upregulated in the shWTAP group are highlighted in red, while downregulated genes are shown in blue. **(C)** GO enrichment analysis for genes differentially downregulated in the shWTAP-treated BMDMs, compared to the shNC group. **(D)** GO enrichment analysis for genes differentially upregulated in the shWTAP-treated BMDMs, compared to the shNC group. **(E)** GSEA of genes expressed in BMDMs treated with shNC and shWTAP. **(F)** Ring heatmap displaying the relative expression levels of osteogenesis-related genes between BMDMs treated with shNC and shWTAP (n = 3 per group).

To achieve a thorough understanding of WTAP’s regulatory roles in macrophage-associated inflammation, we conducted RNA sequencing (RNA-seq) on BMDMs treated with either shNC or shWTAP. Unsupervised principal component analysis (PCA) displayed a distinct separation between the two groups, highlighting significant differences in their gene expression profiles. Within each group, replicates clustered tightly, indicating consistent gene expression profiles across samples (**Figure 3A**). Differential gene expression analysis, depicted through a volcano plot, revealed that shWTAP-treated BMDMs exhibited 1,909 significantly upregulated and 903 significantly downregulated genes compared to the shNC group (**Figure 3B**). GO enrichment analysis showed that downregulated genes in the shWTAP group were primarily pertained to “methylation,” “translation,” and “mRNA processing,” suggesting an impact of WTAP knockdown on macrophage methylation and mRNA modifications (**Figure 3C**). Conversely, upregulated genes were enriched in terms such as “immune system process,” “immune response,” “skeletal system development,” “osteoblast differentiation,” and “bone development,” indicating that WTAP knockdown may augment immune responses and promote osteogenesis in macrophages (**Figure 3D**). GSEA of the differentially expressed genes further indicated that downregulated genes in the shWTAP group are associated with RNA methylation processes, whereas upregulated genes are linked to osteoimmune activities (**Figure 3E**). Notably, genes related to osteogenesis were significantly more expressed in the shWTAP-treated BMDMs than in the shNC group (**Figure 3F**). These findings suggest that WTAP downregulation in macrophages plays a pivotal role in modulating osteoimmune responses and promoting osteogenesis, highlighting potential therapeutic targets for the management of periodontitis.

### 3.4 Enhanced osteogenic differentiation of BMSCs mediated by reduced WTAP expression in macrophages

**Figure 4:**
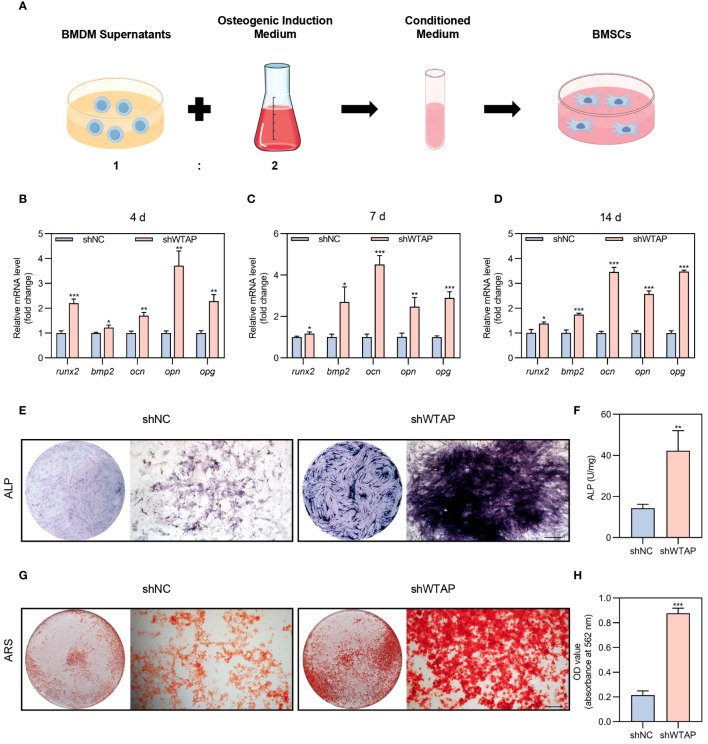
Reducing of WTAP expression in macrophages promotes BMSC osteogenic differentiation. **(A)** Schematic diagram of the indirect co-culture system between BMDMs and BMSCs. **(B-D)** RT-PCR analysis quantifying the mRNA levels of osteogenic markers *runx2*, *bmp2*, *ocn*, *opn* and *opg* in BMSCs co-cultured with conditional medium at 4 **(B)**, 7 **(C)** and 14 **(D)** days (n = 3). **(E)** Representative images of ALP staining of BMSCs cultured in conditional medium for 14 days. Scale bar = 500 μm. **(F)** Quantitative analysis of ALP activity of BMSCs cultured in conditional medium for 14 days (n = 3). **(G)** Representative images of ARS staining of BMSCs cultured in conditional medium for 21 days. Scale bar = 500 μm. **(H)** Quantitative analysis of ARS staining in BMSCs (n = 3). Data are presented as the mean ± SD from at least three independent experiments. *P* values were calculated using two-tailed Student’s t test; *P <0.05, **P <0.01, ***P <0.001.

To investigate the impact of WTAP expression levels in macrophages on the osteogenic differentiation of BMSCs, we utilized conditioned medium from BMDMs treated with either shNC or shWTAP for co-culturing BMSCs (**Figure 4A**). We measured and analyzed the mRNA expression levels of critical osteogenesis-related genes, including *runx2*, *bmp2*, *ocn*, *opn*, and *opg*, in BMSCs co-cultured over periods of 4, 7, and 14 days. Our findings indicated increased mRNA levels in the shWTAP group compared to the shNC group (**Figures 4B–D**). Further, we incubated BMSCs with the conditioned medium for 14 or 21 days and performed ALP and ARS staining to assess osteogenic activity. Quantitative analysis of ALP staining demonstrated that BMSCs in the shWTAP group exhibited higher ALP activity compared to the shNC group, signifying enhanced osteogenic activity (**Figures 4E, F**). Additionally, both qualitative and quantitative assessments from the ARS assay supported these findings. Specifically, the shWTAP group showed a greater number of bone-mineralized nodules, indicating increased osteogenic potential (**Figures 4G, H**). Collectively, these results suggest that reducing WTAP expression in macrophages promotes the osteogenic differentiation of BMSCs.

### 3.5 AAV-mediated silencing of WTAP promotes periodontal tissue regeneration by regulating macrophage polarization

**Figure 5:**
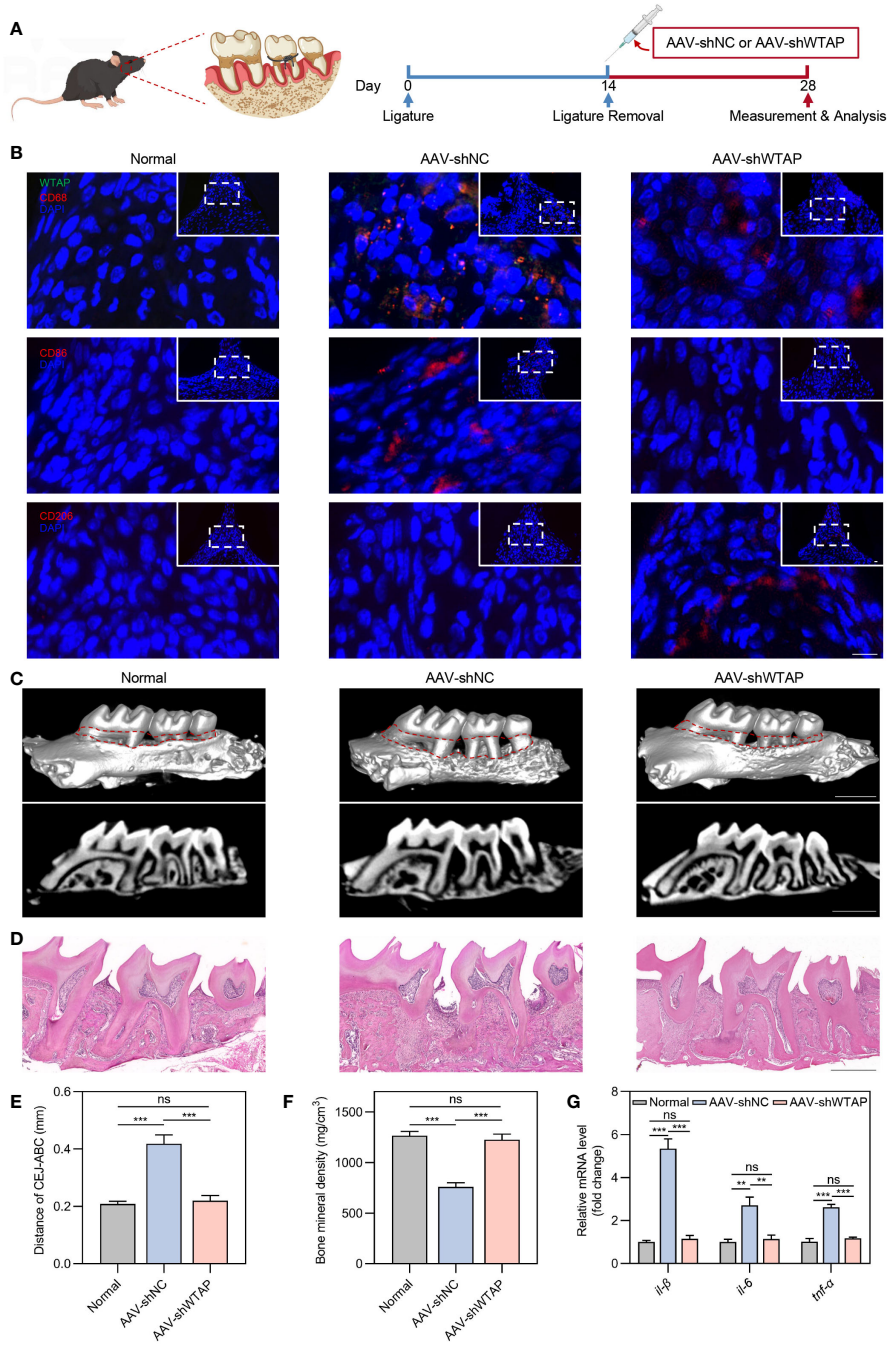
AAV-mediated silencing of WTAP enhances periodontal tissue regeneration by modulating macrophage polarization. **(A)** Schematic illustration of the local injection procedure of AAV-shNC or AAV-shWTAP into the gingival tissues in mice with periodontitis. **(B)** Representative immunofluorescent staining of gingival tissues showing WTAP (green), CD68 (red), CD86 (red), CD206 (red) and nuclei (DAPI, blue). Scale bar = 10 μm. **(C)** Representative micro-CT three-dimensional isosurface and sagittal images displaying the maxillary alveolar bone in normal, AAV-shNC-treated and AAV-shWTAP-treated mice. Scale bar = 1 mm. **(D)** Representative H&E-stained images of maxillary alveolar bone from normal, AAV-shNC-treated and AAV-shWTAP-treated mice. Scale bar = 0.5 mm. **(E)** Quantification of the CEJ-ABC distance in maxillary molars from normal, AAV-shNC-treated and AAV-shWTAP-treated mice (n = 6). **(F)** Quantification of BMD surrounding the second molar from normal, AAV-shNC-treated and AAV-shWTAP-treated mice (n = 6). **(G)** RT-PCR analysis quantifying the mRNA levels of *il-1β*, *il-6* and *tnf-α* in gingival tissues from normal, AAV-shNC-treated and AAV-shWTAP-treated mice (n = 6). Data are presented as the mean ± SD from at least three independent experiments. *P* values were calculated using one-way ANOVA; **P <0.01, ***P <0.001, ns indicates no significant difference.

Next, we explored whether silencing WTAP could enhance periodontal tissue regeneration by promoting macrophage polarization towards the M2 phenotype. To this end, we developed an AAV vector, AAV-shWTAP, designed to specifically silence WTAP expression in macrophages. Following 14 days of ligature placement around the molars of mice, the ligatures were removed, and AAV-shNC or AAV-shWTAP was locally administered into the gingival tissues. Tissue samples were collected for analysis two weeks post-injection (**Figure 5A**). Immunofluorescence analysis of the gingival tissues revealed that, compared to the normal group (healthy mice), the AAV-shNC group exhibited an increase in CD86^+^ macrophages along with elevated WTAP expression within these cells. In contrast, compared to the AAV-shNC group, WTAP expression in macrophages was significantly reduced in the AAV-shWTAP group. This reduction was accompanied by a notable decrease in CD86^+^ macrophages and an increase in CD206^+^ macrophages (**Figure 5B**). These results suggest that AAV-shWTAP treatment effectively downregulated WTAP expression in macrophages, thereby facilitating their transition from an M1 to an M2 phenotype. Furthermore, periodontal bone regeneration in the AAV-shWTAP treated mice was significantly enhanced compared to the AAV-shNC group, approaching levels seen in normal mice. This improvement was evidenced by decreased cemento-enamel junction-alveolar bone crest (CEJ-ABC) distance and increased BMD (**Figures 5C–F**). Additionally, we observed that mRNA levels of pro-inflammatory cytokines (*il-1β*, *il-6*, and *tnf-α*) in the gingival tissues of AAV-shWTAP mice were significantly reduced compared to those in the AAV-shNC group, nearing levels observed in the normal group (**Figure 5G**). Collectively, our findings demonstrate that AAV-mediated silencing of WTAP promotes periodontal tissue regeneration by regulating macrophage polarization, potentially offering a novel therapeutic approach for managing this chronic inflammatory disease.

## 4 Discussion

Here, we delved into the role of WTAP in periodontal immunomodulation and evaluated its potential as a therapeutic target for managing periodontitis. Our findings indicate that periodontitis leads to the upregulation of WTAP in macrophages, a pivotal change that significantly influences their polarization. Specifically, the knockdown of WTAP facilitates the transition from the pro-inflammatory M1 phenotype to the anti-inflammatory M2 phenotype, thereby enhancing the osteogenic differentiation of BMSCs. The local administration of AAV-shWTAP effectively promotes periodontal tissue regeneration by regulating macrophage polarization, highlighting the therapeutic potential of targeting WTAP in periodontitis treatment.

Periodontitis involves a complex interplay between microbial dysbiosis and the host’s immune system, where disease progression is primarily driven by immune responses rather than merely the presence of microbial agents (5, 22). This emphasizes the importance of focusing on immunomodulation in the treatment of periodontitis (22). Recent literatures underscore the critical role of epigenetic modifications in determining the destiny and functionality of immune cells (23–26). Prior studies have pointed out the vital importance of m^6^A modifications in shaping the diversity and complexity of the immune microenvironment in periodontitis (27, 28). In alignment with these studies, our research confirmed that WTAP, as a key m^6^A methyltransferase, plays an instrumental role in regulating immune homeostasis in the periodontal region. Our findings further illuminated the potential of m^6^A modifications to modulate the periodontal immune microenvironment, offering new perspectives on the therapeutic applications of these modifications in periodontitis.

Macrophages play a central role in both the destructive and reparative phases of periodontitis, with their phenotypic heterogeneity offering new avenues for therapeutic intervention (8). The transition of macrophages from the M1 to the M2 phenotype is a crucial aspect of current immune therapeutic strategies aimed at fostering periodontal regeneration (8, 29). Several studies have identified potential targets such as PPARγ (30), ROS (31), Akt2 (32), and RGS12 (33), which significantly influence macrophage function and the progression of periodontal disease. However, due to the complexity and dynamic nature of macrophage polarization, influenced by various factors, further research is needed to fully uncover the mechanisms affecting macrophage function and polarization. Emerging studies highlight the significant influence of m^6^A modifications in regulating macrophage polarization and functionality (12, 14, 34–36). As a functional component of the METTL3-METTL14 m^6^A methyltransferase complex, the role of WTAP in modulating macrophage behavior under inflammatory conditions is yet to be elucidated. Our findings confirmed that knockdown of WTAP promotes a shift from M1 to M2 phenotype under inflammatory conditions, thereby enhancing the osteogenic differentiation of BMSCs, suggesting that WTAP could serve as a novel target for periodontitis treatment. The detailed mechanisms through which WTAP influences this shift require further investigation and represent an important area for future research.

Advances in gene therapy, particularly using AAV platforms, have enhanced this approach by providing a means to deliver sustained and precise gene modulation directly at the site of disease (37, 38). AAV-based gene silencing has emerged as a powerful strategy within clinical research, targeting a range of pathological disorders with high specificity and minimal invasiveness (39, 40). These therapies work by incorporating specific genes into the genome, thereby correcting or compensating for dysfunctional gene expression (39). The utility of AAV in achieving long-term gene silencing through local administration makes it particularly appealing for diseases like periodontitis, where localized treatment can directly address tissue-specific pathology without systemic effects (41, 42). Our research has employed AAV-mediated silencing of WTAP in macrophages to modulate osteoimmune responses during periodontitis. By specifically silencing the WTAP gene in macrophages, our AAV-shWTAP strategy has demonstrated efficacy in mitigating periodontal tissue damage and promoting alveolar bone regeneration. This approach underscores the potential of AAV-shWTAP as a therapeutic intervention not only for periodontitis but also potentially for other inflammatory diseases involving WTAP, such as hepatocellular carcinoma (16) and osteoarthritis (19). Moreover, future exploration of small molecule drugs that can inhibit WTAP may offer additional therapeutic potential.

Our findings demonstrate that WTAP inhibition shifts macrophage polarization to the anti-inflammatory M2 phenotype, enhancing osteogenic responses in BMSCs and fostering tissue regeneration. This pivotal role of WTAP positions it as a promising therapeutic target for periodontitis, with potential applications in gene therapy and adjunctive treatments. *In vivo* silencing of WTAP with AAV-shWTAP further validates its potential to improve periodontal outcomes, underscoring WTAP as a promising target for gene therapy in periodontitis. Specifically targeting WTAP in macrophages using AAV-shWTAP or similar gene-editing technologies could become a cornerstone of periodontitis treatments. This approach offers a new avenue for patients who do not respond well to conventional therapies and minimizes potential side effects associated with systemic treatments. Additionally, the development of WTAP inhibitors could serve as adjunctive treatments alongside existing periodontal therapies, particularly in severe or refractory cases of periodontitis, to augment their efficacy.

In conclusion, our study provides compelling evidence of the critical role of WTAP in macrophage-mediated osteoimmune responses within the context of periodontitis. Identifying WTAP as a potential therapeutic target opens new avenues for the development of targeted immunotherapeutic interventions aimed at mitigating the inflammatory processes in periodontitis.

## Data availability statement

The datasets presented in this study can be found in online repositories. The names of the repository/repositories and accession number(s) can be found below: GSE265968 (GEO).

## Ethics statement

The animal study was approved by the Ethics Committee of Peking University Health Science Center. The study was conducted in accordance with the local legislation and institutional requirements.

## Author contributions

YML: Writing – review & editing, Writing – original draft, Visualization, Investigation, Formal analysis, Conceptualization. YY: Writing – review & editing, Writing – original draft, Visualization, Investigation, Formal analysis, Conceptualization. YN: Writing – original draft, Investigation, Formal analysis. YL: Writing – original draft, Investigation. ZH: Writing – original draft, Investigation. SS: Writing – original draft, Investigation. YC: Writing – original draft, Formal analysis. BH: Writing – original draft, Formal analysis. YH: Writing – review & editing, Writing – original draft, Supervision, Funding acquisition, Conceptualization. XD: Writing – review & editing, Writing – original draft, Supervision, Funding acquisition, Conceptualization.
