# Supplementary material for: The role of WTAP in regulating macrophage-mediated osteoimmune responses and tissue regeneration in periodontitis
Source: Front Immunol. 2024 Jul 16;15:1423378. doi: 10.3389/fimmu.2024.1423378 (PMC11286459; doi:10.3389/fimmu.2024.1423378)
Supplement: Supplementary file 1 [file DataSheet_1.pdf]

Supplementary Material

Supplementary Table 1. The primers used for quantitative RT-PCR .

Gene	Primer sequence (5' to 3') -Forward	Primer sequence (5' to 3')-Reverse
gapdh	CCATCACCATCTTCCAGG	AGACTCCACGACATACTCA
wtap	AAACTGGAACAAGCCCAA	CCTGGATAAGCATTCGACA
inos	GCACCGAGATTGGAGTTC	GAGCACAGCCACATTGAT
il-10	GACAACATACTGCTAACC	AGGTAAAACTGGATCATT
il-12	ATGTGGAATGGCGTCTCTG	GCTGGTGCTGTAGTTCTCAT
il-6	GAGAGGAGACTTCACAGA	GCATCATCGTTGTTCATAC
tnf-α	CAACGGCATGGATCTCAA	AAATCGGCTGACGGTGTG
mrc-1	GGACTCTGGATTGGACTC	GGCTCTGATGATGGACTT
fizz-1	GTAGCAGTCATCCCAGCAG	TCTTGCCAATCCAGCTAAC
ym-1	GACTTGCGTGACTATGAA	CTGAGGAGTAGAGACCAT
runx2	CCACGGCCCTCCCTGAACTCT	ACTGGCGGGGTGTAGGTAAAGGTG
bmp2	GGACCCGCTGTCTTCTAGTG	TTCCTCGATGGCTTCTTCGT
ocn	TGAGGTCAGAGAGACAGAG	ACAGACAAGTCCCACACA
opn	GGACCTCACCTCTCACAT	TTGGAATGCTCAAGTCTGT
opg	ACCCAGAAACTGGTCATCAGC	CTGCAATACACACACTCATCACT
il-1β	CAACCAACAAGTGATATTCTCC	TGCCGTCTTTCATTACACAG

Supplementary Figures

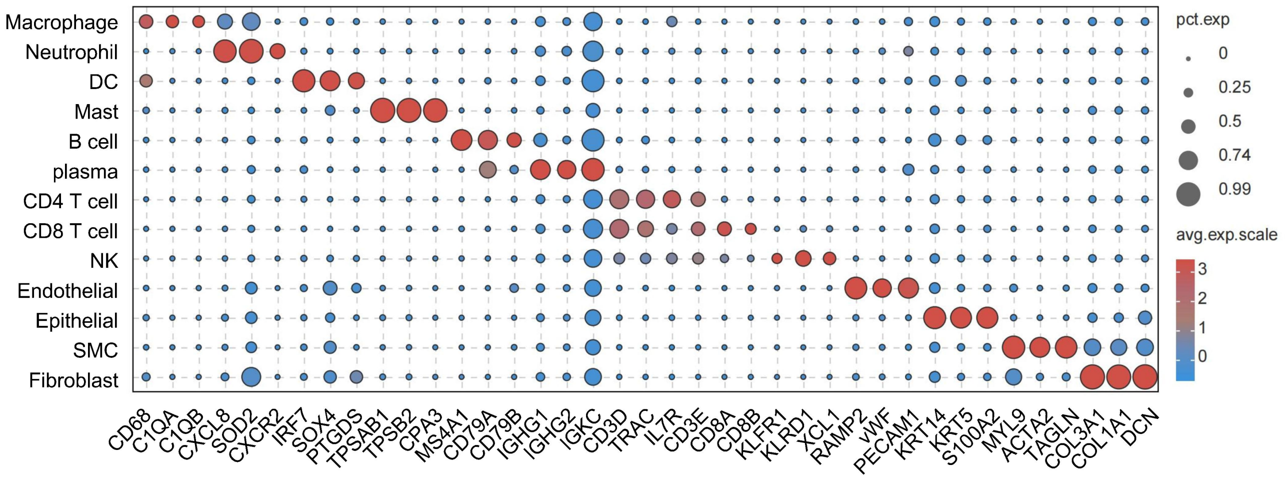

Supplementary Figure 1. Dot plot showing the espression of known cell-type-enriched marker genes across 13 cell clusters. Dot color represents average expression level; dot size indicates the percentage of cells expressing the gene.

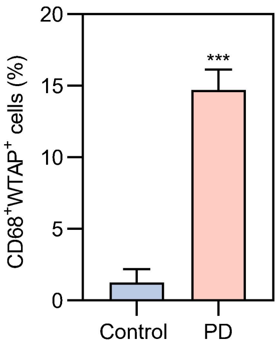

Supplementary Figure 2. Quantitative analysis of the percentage of $CD68^{+}WTAP^{+}$ cells in gingival tissue sections from control and periodontitis (PD) mice. Data are presented as the mean ± SD from at least three independent experiments. *P* values were calculated using two-tailed Student's t test; ****P* <0.001.

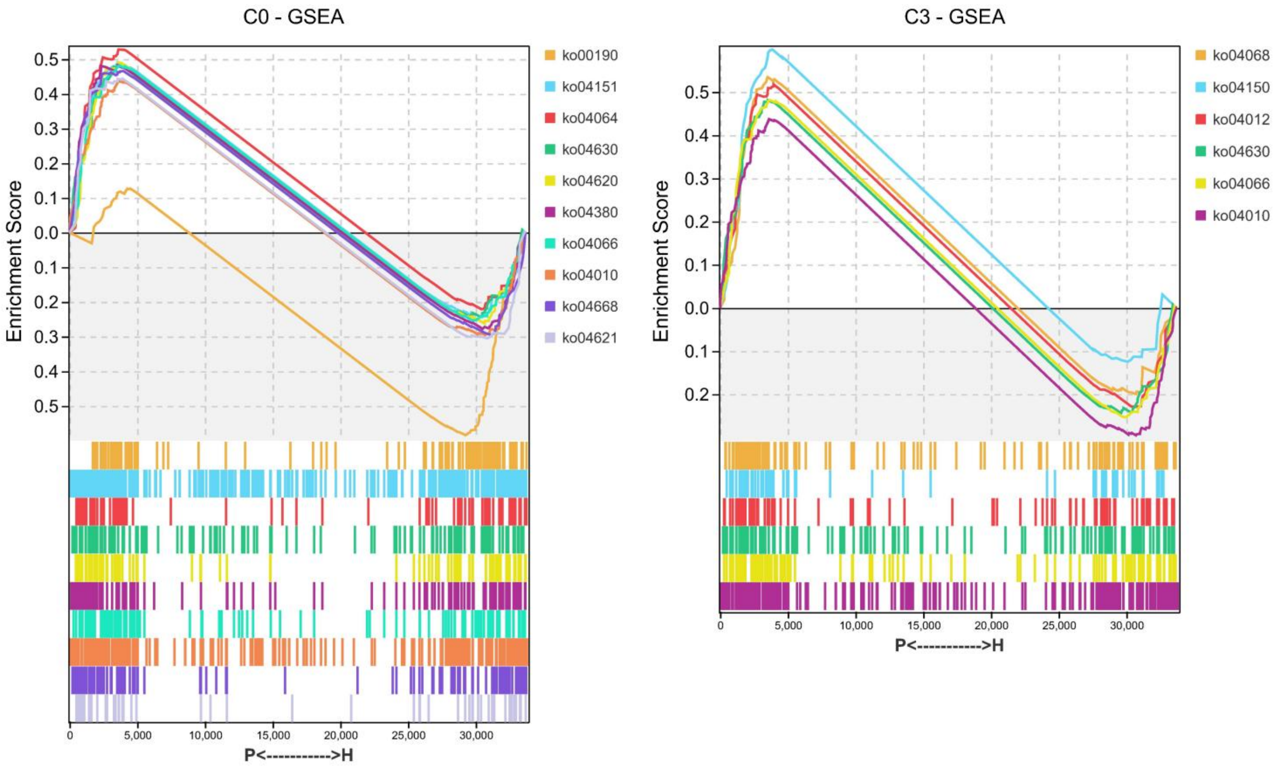

Supplementary Figure 3. GSEA of genes expressed in macrophage subclusters C0 and C3 of patients with periodontitis (P) versus healthy subjects (H). ko00190: Oxidative phosphorylation; ko04151: PI3K-Akt signaling pathway; ko04064: NF-kappa B signaling pathway; ko04630: JAK-STAT signaling pathway; ko04620: Toll-like receptor signaling pathway; ko04380: Osteoclast differentiation; ko04066: HIF-1 signaling pathway; ko04010: MAPK signaling pathway; ko04668: TNF signaling pathway; ko04621: NOD-like receptor signaling pathway; ko04068: FoxO signaling pathway; ko04150: mTOR signaling pathway; ko04012:ErbB signaling pathway.

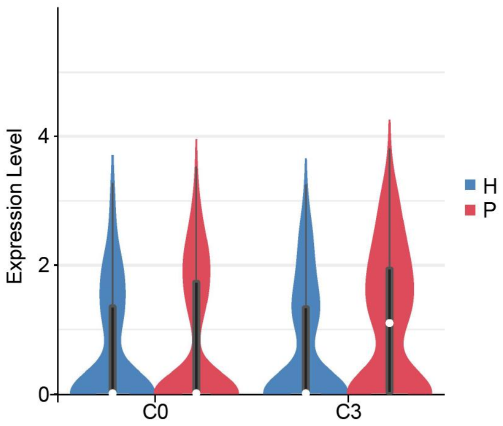

Supplementary Figure 4. Violin plot illustrating the expression levels of WTAP in macrophage subclusters C0 and C3, as determined by scRNA-seq data from patients with periodontitis (P) and healthy controls (H).

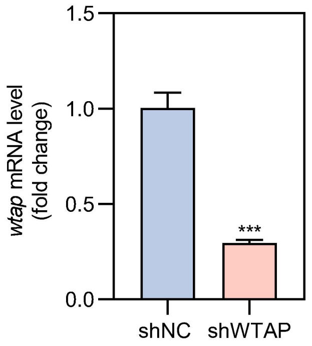

Supplementary Figure 5. RT-PCR analysis quantifying the mRNA levels of *wtap* in BMDMs from the shNC and shWTAP groups (n = 3). Data are presented as the mean ± SD from at least three independent experiments. *P* values were calculated using two-tailed Student's t test; ****P* <0.001.

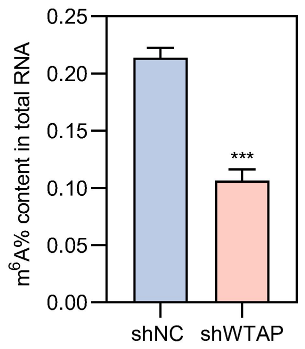

Supplementary Figure 6. Comparison of m^6A methylation levels in BMDMs treated with shNC and shWTAP following 24 hours of LPS induction (n = 3). Data are presented as the mean ± SD from at least three independent experiments. *P* values were calculated using two-tailed Student's t test; ****P* <0.001.

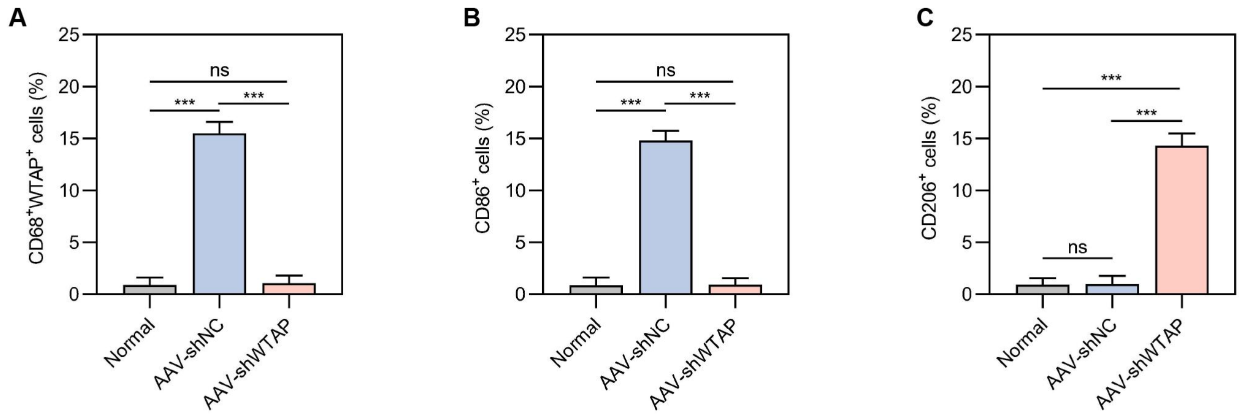

Supplementary Figure 7. Quantitative analysis of the percentage of $CD68^{+}WTAP^{+}$ cells (A), $CD86^{+}$ cells (B), and $CD206^{+}$ cells (C) in gingival tissue sections from normal, AAV-shNC-treated and AAV-shWTAP-treated mice. Data are presented as the mean ± SD from at least three independent experiments. *P* values were calculated using one-way ANOVA; ****P* <0.001, ns indicates no significant difference.
